# Is Baseline PET/CT-Derived Metabolic Tumor Burden Associated with Treatment Response and Survival in Hodgkin Lymphoma?

**DOI:** 10.3390/jcm15176686

**Published:** 2026-08-28

**Authors:** Büşra Tuğçe Tonyalı, Mehmet Günhan Tekin, Sefa Bayram, Selin Gül Yaran, Gökhan Burul, Edibe Sevde Eker, Tahir Alper Cinli, Hasan Göze, Burcu Esen Akkaş, Mesut Ayer

**Affiliations:** 1 Department of Hematology, Başakşehir Çam and Sakura City Hospital, Istanbul 34480, Türkiyemesutayerdr@hotmail.com (M.A.); 2 Department of Nuclear Medicine, Başakşehir Çam and Sakura City Hospital, Istanbul 34480, Türkiye; sefabayram38@gmail.com (S.B.);

**Keywords:** Hodgkin lymphoma, PET/CT, metabolic tumor volume, total lesion glycolysis, treatment response, progression-free survival, overall survival

## Abstract

**Background/Objectives:** Metabolic tumor volume (MTV) and total lesion glycolysis (TLG) provide quantitative assessments of metabolically active tumor burden; however, their prognostic value in Hodgkin lymphoma (HL) remains uncertain. This study evaluated the associations of baseline PET/CT-derived MTV and TLG with clinical disease characteristics, treatment response, progression-free survival (PFS), and overall survival (OS) in patients with classical HL. **Methods:** This retrospective single-center study included 105 adults diagnosed with classical HL between July 2020 and April 2023. Baseline PET/CT images were analyzed using LIFEx 7.1.0. Volumes of interest were delineated using a fixed threshold of 41% of SUVmax. Total MTV and TLG were calculated by summing the values of all FDG-avid lesions. Survival outcomes were evaluated using Kaplan–Meier analysis, the log-rank test, and Cox proportional hazards regression. **Results:** The median age was 33 years, and the median follow-up was 24 months. Higher MTV and TLG were significantly associated with advanced-stage disease, high International Prognostic Score, B symptoms, bulky disease, and extranodal involvement. Higher MTV was associated with a lower end-of-treatment response rate, whereas higher TLG was associated with lower interim and end-of-treatment response rates. Disease progression occurred in 19 patients, and 11 patients died. Compared with patients with MTV < 249, those with MTV ≥ 249 had numerically higher hazards of progression (HR = 2.18, 95% CI: 0.88–5.40; *p* = 0.09) and death (HR = 1.21, 95% CI: 0.37–3.96; *p* = 0.75). Compared with patients with TLG < 1252, those with TLG ≥ 1252 had a numerically higher hazard of progression (HR = 1.53, 95% CI: 0.61–3.86; *p* = 0.37) but a lower hazard of death (HR = 0.56, 95% CI: 0.17–1.85; *p* = 0.34). However, all confidence intervals included 1, and none of these differences reached statistical significance. **Conclusions:** Baseline MTV and TLG reflect disease burden and are associated with treatment response in classical HL. However, neither parameter was significantly associated with PFS or OS in this cohort. Their potential value as survival prognostic biomarkers requires confirmation in larger prospective studies with longer follow-up.

## 1. Introduction

Hodgkin lymphoma (HL) is a highly curable hematologic malignancy, with long-term survival achieved in the majority of patients through modern treatment strategies [1,2]. However, a percentage of patients develop primary refractory or relapsed disease, highlighting the need for accurate risk stratification and individualized treatment approaches. ^18^F-fluorodeoxyglucose (FDG) positron emission tomography (PET/CT) has become the standard imaging modality for disease staging and treatment response assessment using the Deauville scoring system [3]. Although several studies have suggested that baseline PET/CT provides valuable prognostic information [4], conventional imaging assessment may not adequately quantify tumor burden or reflect the biological heterogeneity of the disease.

In recent years, increasing attention has been focused on quantitative PET/CT biomarkers that may provide additional prognostic information at diagnosis beyond conventional imaging assessment and complement the predictive value of the Deauville score. Among these, metabolic tumor volume (MTV) and total lesion glycolysis (TLG) have emerged as promising biomarkers for improving risk stratification and predicting clinical outcomes in lymphoma [5,6,7,8,9]. By providing a more comprehensive assessment of tumor burden and disease dissemination, these parameters may facilitate more accurate prognostic assessment and support individualized treatment strategies.

However, the prognostic value of MTV and TLG in HL remains controversial. Reported findings are heterogeneous owing to differences in segmentation methods, threshold values, patient populations, and treatment protocols across studies [10,11,12,13]. Furthermore, standardized cutoff values for the routine clinical use of these parameters have not yet been established.

## 2. Materials and Methods

In the present study, we aimed to evaluate the association between baseline PET/CT-derived MTV and TLG and treatment response and survival outcomes in patients with HL in a single-center cohort.

Patients aged ≥ 18 years who were diagnosed with classical HL between July 2020 and April 2023 were retrospectively enrolled. Eligibility criteria included no previous history of malignancy, no prior chemotherapy or radiotherapy, and the presence of at least two FDG-avid lesions on baseline PET/CT.

All patients received two cycles of ABVD (doxorubicin [adriamycin], bleomycin, vinblastine, and dacarbazine), followed by interim PET/CT assessment. Patients with a Deauville score of 1–3 continued treatment with AVD, whereas those with a Deauville score of 4–5 were switched to escalated BEACOPP (bleomycin, etoposide, doxorubicin, cyclophosphamide, vincristine, procarbazine, and prednisone). Radiotherapy was administered to patients with residual disease when clinically indicated.

[18F]FDG PET/CT acquisitions and the accompanying image reconstructions were carried out in accordance with the European Association of Nuclear Medicine (EANM) recommendations for oncological imaging [14]. All studies were acquired on an Ingenuity TF 64 system (Philips Medical Systems, Inc., Cleveland, OH, USA). Prior to the examination, patients were required to have fasted for a minimum of six hours, and serum glucose was checked immediately before tracer administration; the radiopharmaceutical was given at the appropriate activity only to those whose glucose values remained under 200 mg/dL. Subjects then rested in a quiet, isolated room for 45–60 min to permit adequate tracer distribution and uptake. After a topogram was obtained with the patient supine, covering the region from the vertex to the proximal thighs, a low-dose CT (113 mAs, 120 kV, 4 mm slice thickness) was acquired for attenuation correction. PET data were subsequently collected over the identical field of view in the caudocranial direction. Reconstructed datasets included maximum intensity projection (MIP) images together with transverse, coronal and sagittal sections.

Baseline PET/CT images were analyzed using LIFEx software (version 7.1.0; LIFEx, CEA, Orsay, France). For each FDG-avid lesion, a volume of interest (VOI) was delineated using a fixed threshold of 41% of the lesion-specific SUVmax, in accordance with the isocontour approach described in the EANM procedure guidelines for tumor imaging [14]. This method was selected because it has demonstrated good volumetric accuracy and interobserver reproducibility in lymphoma and has been widely used in previous studies evaluating baseline metabolic tumor burden in Hodgkin lymphoma [12]. All automatically generated contours were visually reviewed and manually corrected when necessary. Physiological uptake was excluded, while bone marrow and splenic uptake were included only when focal involvement was present. MTV was calculated for each lesion, and total metabolic tumor volume (tMTV) was obtained by summing the MTV values of all lesions. Total lesion glycolysis (tTLG) was calculated as the sum of lesion-level TLG values, with each lesion-level TLG defined as MTV × SUVmean.

Treatment response was assessed according to the Lugano classification [15]. Progression-free survival (PFS) was defined as the time from diagnosis to disease progression, relapse, or death from any cause. Overall survival (OS) was defined as the time from diagnosis to death from any cause or the date of the last follow-up.

Statistical analyses were performed using IBM SPSS Statistics version 25.0 (IBM Corp., Armonk, NY, USA) and MedCalc version 23.0.3 (MedCalc Software Ltd., Ostend, Belgium). Time-dependent receiver operating characteristic (ROC) curve analysis was performed at 24 months using the inverse probability of censoring weighting (IPCW) method to account for censored survival data. Continuous variables were compared using Student’s *t* test or the Mann–Whitney U test according to data distribution. Relationships between categorical variables were evaluated using the chi-square test statistic; when expected cell frequencies were low, the exact significance values of the chi-square test were used. Differences between two proportions were evaluated using the Z-test statistic.

Survival analyses were performed using the Kaplan–Meier method, and survival curves were compared using the log-rank test. Factors associated with survival were evaluated using Cox proportional hazards regression, and hazard ratios (HRs) with 95% confidence intervals (CIs) were reported. The proportional hazards assumption was assessed for all Cox regression models of PFS and OS using the Grambsch–Therneau test based on scaled Schoenfeld residuals. A two-sided *p* value < 0.05 was considered statistically significant.

## 3. Results

A total of 105 patients with classical HL were included in the study. The median age was 33 years (range, 18–82 years), and 63 patients (60.0%) were male. According to the IPS, 70 patients (66.7%) were classified as low-risk (IPS 0–3), whereas 35 (33.3%) were classified as high-risk (IPS ≥ 4). Nodular sclerosis was the predominant histological subtype (64.8%), followed by mixed cellularity (27.6%). The interim response rate was 93.9%, and the end-of-treatment response rate was 89.1%. Disease progression occurred in 19 patients (18.1%), while 11 patients (10.5%) died during follow-up. The median follow-up duration was 24 months (range, 2–64 months).

Interim PET response was evaluable in 99 of the 105 patients (94.3%). It could not be evaluated in six patients (5.7%): three patients died before the interim PET assessment, and three underwent interim PET/CT at external centers, but the corresponding results were unavailable. End-of-treatment response was evaluable in 92 patients (87.6%) and unavailable in 13 patients (12.4%): six patients died before the end-of-treatment assessment, and seven underwent PET/CT at external centers, but their results could not be retrieved. All patients, including those who died before the response assessments, were retained in the survival analyses, and the deaths were included as events in the OS analysis.

The demographic, clinical, and treatment characteristics of the study population are summarized in Table 1.

In the time-dependent ROC analysis performed at a 24-month time horizon using the IPCW method, the AUC values for OS were 0.480 (95% CI: 0.333–0.641; *p* = 0.804) for MTV and 0.491 (95% CI: 0.324–0.653; *p* = 0.914) for TLG, with sensitivity/specificity values of 89.2%/36.7% and 100.0%/16.3% at the identified cutoffs, respectively. For PFS, the AUC values were 0.568 (95% CI: 0.427–0.705; *p* = 0.346) for MTV and 0.541 (95% CI: 0.404–0.682; *p* = 0.570) for TLG, with corresponding sensitivity/specificity values of 60.5%/64.4% and 73.0%/48.9%, respectively. Overall, neither MTV nor TLG demonstrated statistically significant discriminatory performance for 24-month OS or PFS (all *p* > 0.05). Therefore, median values were used as cutoff points for subsequent analyses.

Patients were stratified into two groups according to the median MTV value. Compared with the low-MTV group, patients with high MTV had significantly higher rates of advanced-stage disease (particularly stage IV), high IPS scores, B symptoms, bulky disease, and extranodal involvement (all *p* < 0.001). In addition, the end-of-treatment response rate was significantly lower in the high-MTV group (*p* = 0.04) (Table 2).

Patients were stratified into two groups according to the median TLG value. Compared with the low-TLG group, patients with high TLG had significantly higher rates of advanced-stage disease, particularly stage IV disease (*p* = 0.004), high IPS scores, B symptoms, bulky disease, and extranodal involvement (all *p* < 0.001). In addition, both interim and end-of-treatment response rates were significantly lower in the high-TLG group (Table 3).

**Figure 1:**
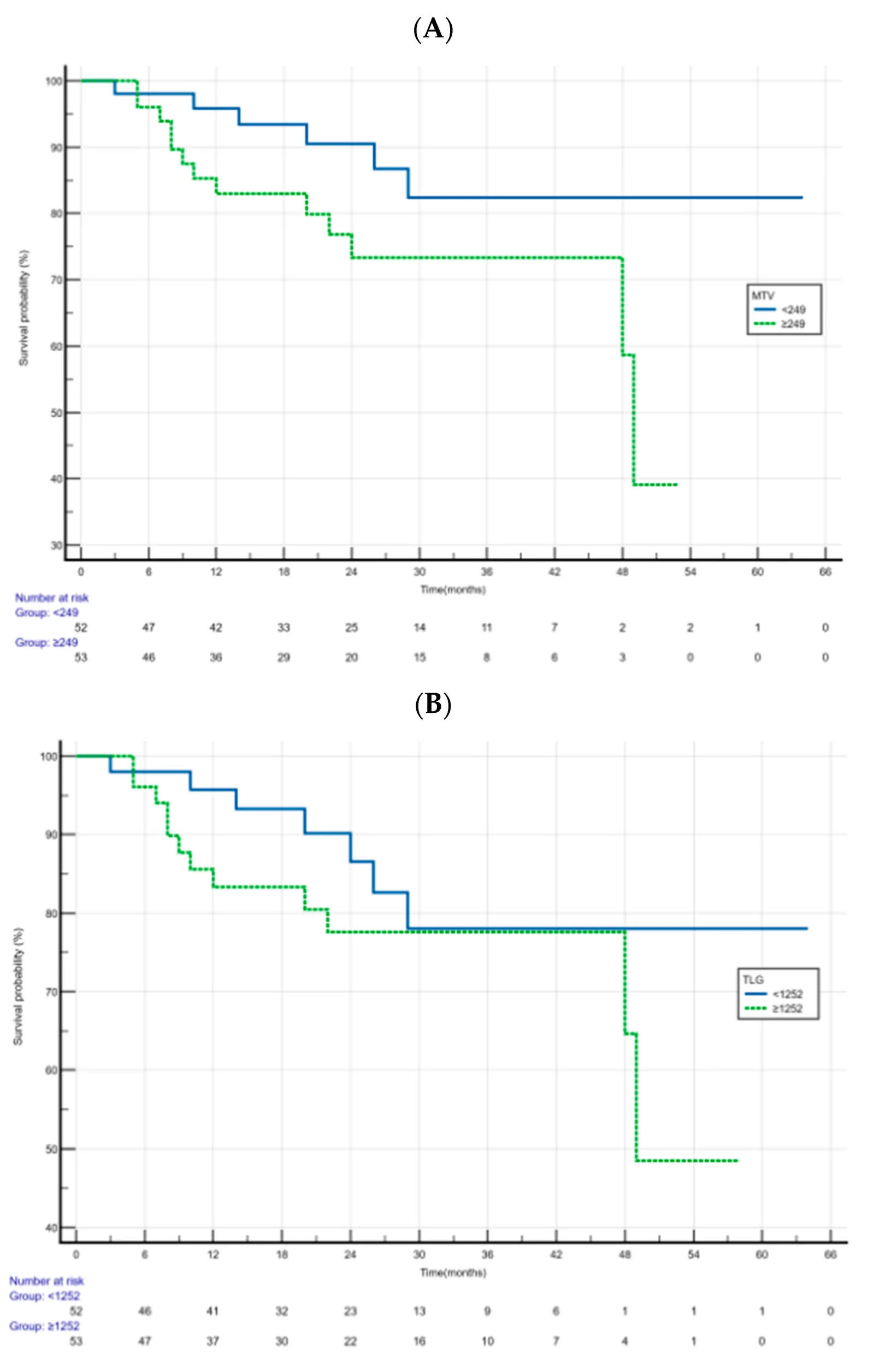
Kaplan–Meier curves for PFS. Kaplan–Meier analysis showed longer PFS in patients with MTV < 249 (**A**) and TLG < 1252 (**B**); however, these differences were not statistically significant (log-rank *p* = 0.09 and *p* = 0.37, respectively).

Disease progression occurred in 19 of the 105 patients (18.1%). The estimated mean progression-free survival (PFS) for the entire cohort was 49 months (95% CI, 43–56 months). Patients with MTV ≥ 249 had a 2.18-fold higher risk of disease progression than those with MTV < 249 (HR, 2.18; 95% CI, 0.88–5.40; *p* = 0.09). Similarly, patients with TLG ≥ 1252 had a 1.53-fold higher risk of disease progression than those with TLG < 1252 (HR, 1.53; 95% CI, 0.61–3.86; *p* = 0.37). However, neither association reached statistical significance (Table 4). For the Cox regression models of PFS, no evidence of violation of the proportional hazards assumption was detected for MTV (χ^2^ = 0.207; *p* = 0.649), or TLG (χ^2^ = 1.470; *p* = 0.225). The corresponding Kaplan–Meier PFS curves according to MTV and TLG are presented in Figure 1.

**Figure 2:**
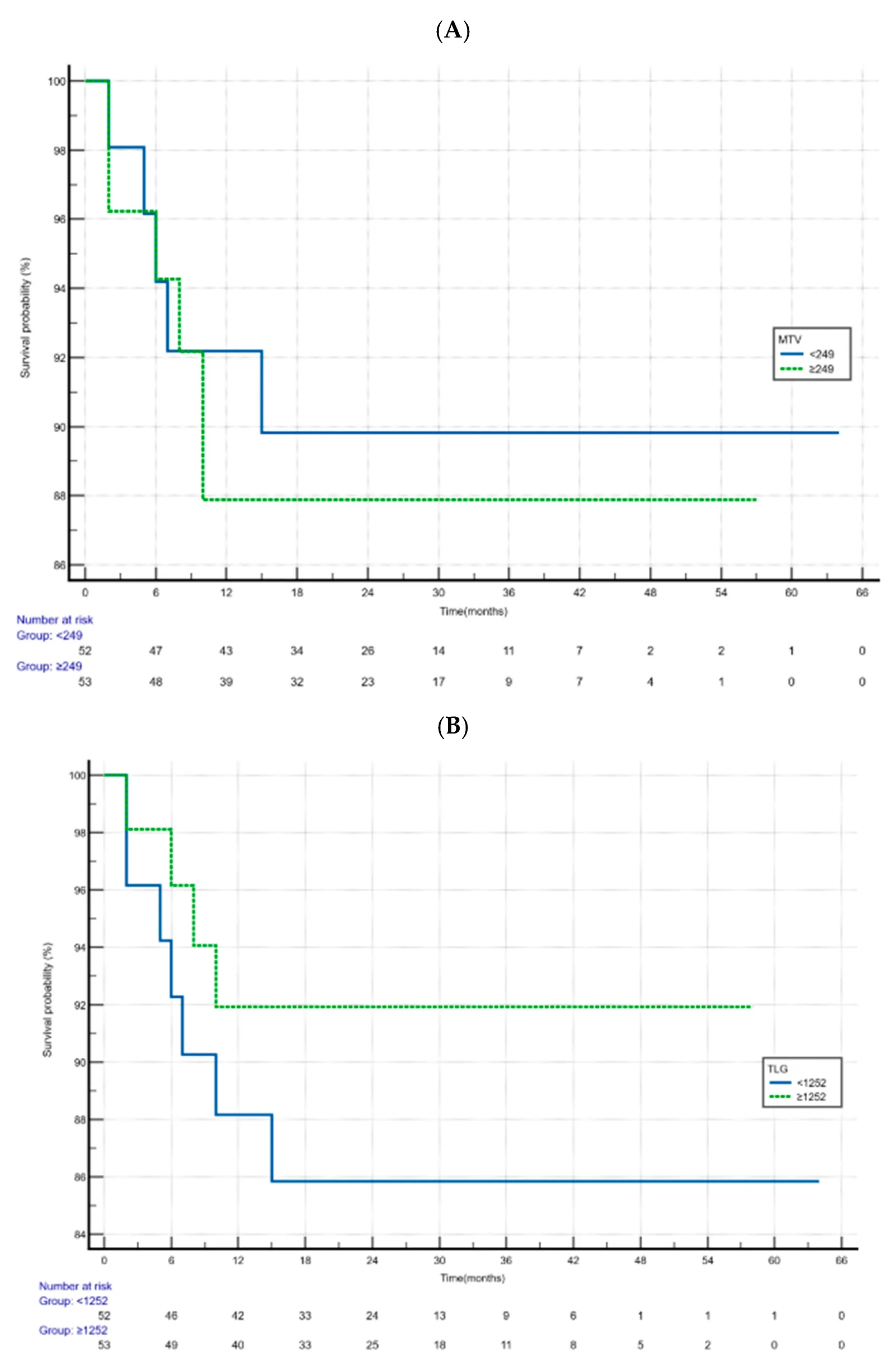
Kaplan–Meier curves for OS according to (**A**) MTV and (**B**) TLG. Kaplan–Meier analysis showed a higher probability of OS in patients with MTV < 249 (**A**) and TLG < 1252 (**B**). However, the differences between the groups were not statistically significant (log-rank *p* = 0.75 and *p* = 0.34, respectively).

A total of 11 of the 105 patients (10.5%) died during follow-up. The estimated mean OS for the entire cohort was 58 months (95% CI, 54–61 months). Patients with MTV ≥ 249 had a 1.21-fold higher risk of death than those with MTV < 249 (HR, 1.21; 95% CI, 0.37–3.96; *p* = 0.75). Patients with TLG ≥ 1252 had a lower estimated hazard of death than those with TLG < 1252 (HR = 0.56; 95% CI: 0.17–1.85; *p* = 0.34); however, this difference was not statistically significant. However, neither association reached statistical significance (Table 5). For the Cox regression models of OS, no evidence of violation of the proportional hazards assumption was detected for MTV (χ^2^ = 0.415; *p* = 0.520) or TLG (χ^2^ = 0.022; *p* = 0.883). The corresponding Kaplan–Meier OS curves according to MTV and TLG are presented in Figure 2.

In parsimonious multivariable Cox regression models adjusted for categorical IPS, neither continuous MTV nor continuous TLG was independently associated with survival outcomes. For OS, the HR was 0.793 for MTV (95% CI: 0.603–1.042; *p* = 0.096) and 0.774 for TLG (95% CI: 0.539–1.113; *p* = 0.167). An IPS of ≥3 was significantly associated with mortality in the MTV model (HR = 4.141, 95% CI: 1.041–16.475; *p* = 0.044), but not in the TLG model (HR = 3.165, 95% CI: 0.855–11.719; *p* = 0.085). For PFS, neither MTV (HR = 0.990, 95% CI: 0.891–1.101; *p* = 0.858) nor TLG (HR = 0.966, 95% CI: 0.791–1.181; *p* = 0.737) was independently associated with progression. Categorical IPS was also not significant in either PFS model.

## 4. Discussion

In the present study, we evaluated the prognostic value of baseline PET/CT-derived MTV and TLG in relation to disease burden, treatment response, and survival outcomes in patients with HL. The main findings of our study were that higher MTV and TLG values were significantly associated with advanced-stage disease, bulky disease, extranodal involvement, B symptoms, and high IPS. In addition, higher MTV was associated with a lower end-of-treatment response rate, whereas higher TLG was associated with both lower interim and end-of-treatment response rates. In the high-MTV and high-TLG groups, none of the associations with PFS or OS reached statistical significance.

The prognostic value of baseline PET/CT-derived MTV and TLG for PFS and OS in HL remains controversial [13]. Several studies have reported that higher MTV and TLG values are associated with shorter PFS and that MTV, in particular, may serve as an independent prognostic biomarker [11,12,16]. In contrast, a recently published study including 98 patients with classical HL reported no significant association between baseline MTV or TLG and PFS, whereas only metabolic changes assessed on interim PET were predictive of outcome [17]. Similarly, Pinochet et al. reported that baseline MTV was associated with PFS but had a more limited impact on OS, suggesting that modern salvage therapies could attenuate the long-term prognostic impact of baseline metabolic tumor burden [18]. In our study, the wide 95% confidence intervals for the PFS and OS hazard ratios, all of which included 1.0, together with the non-significant *p*-values, indicate substantial uncertainty regarding the magnitude and direction of these associations. This uncertainty likely reflects the relatively short median follow-up and limited statistical power resulting from the small number of progression and death events.

In the absence of externally validated clinical thresholds, the median values of MTV (249 mL) and TLG (1252) were used to create two approximately balanced groups for descriptive comparisons. This outcome-independent approach avoided selecting cut-off values that maximized survival differences within the same dataset. Nevertheless, median-based dichotomization results in some loss of quantitative information, and the thresholds obtained are specific to the present cohort rather than clinically validated decision limits. Previous studies have used heterogeneous methods to define high and low MTV or TLG, including receiver operating characteristic analysis, minimum *p*-value approaches, and median-based categorization. The systematic review and meta-analysis by Pak et al. demonstrated considerable variation in cut-off determination across studies, while Dholakia et al. also used median values to categorize PET-derived parameters [19,20]. Direct comparison of absolute thresholds remains difficult because MTV and TLG values are influenced by differences in patient populations, disease characteristics, image acquisition, segmentation methods, and treatment protocols. Therefore, the median values used in this study should be interpreted as cohort-specific exploratory thresholds and should not be directly adopted for routine clinical decision-making without external validation.

Our findings also demonstrated that elevated MTV and TLG were significantly associated with stage IV disease, B symptoms, bulky disease, high IPS, and extranodal involvement. These findings are consistent with previous reports suggesting that volumetric PET parameters reflect both the anatomical extent of disease and its biological aggressiveness [12,16].

In our study, baseline MTV was not significantly associated with interim treatment response but was significantly associated with end-of-treatment response. In contrast, TLG was significantly associated with both interim and end-of-treatment responses. These findings suggest that TLG, which reflects both tumor volume and metabolic activity, may be a more sensitive biomarker for predicting early treatment response than MTV, which reflects only the metabolically active tumor volume. Nevertheless, most previous studies have reported significant associations between baseline MTV and TLG and interim treatment response. Therefore, the discrepancy observed in our study may be explained by differences in patient characteristics, segmentation methods, cutoff determination, and the relatively limited sample size [6,16,21].

The main limitations of this study are its retrospective design and single-center nature. The limited number of events in the current study restricted the ability to evaluate the independent effects of MTV and TLG beyond established clinical prognostic factors such as Ann Arbor stage, IPS, large tumor, B symptoms, and extranodal involvement within a comprehensive multivariate Cox regression model. Additionally, the short follow-up period and limited number of events may have reduced the statistical power of other survival analyses. Furthermore, volumetric parameters derived from PET/CT were evaluated only in conjunction with IPS, and comparative analyses were not performed using other established clinical risk stratification systems such as EORTC or GHSG criteria. Future prospective, multicenter studies with larger patient cohorts and longer follow-up periods are needed to determine whether additional PET-derived parameters such as Dmax and MTV/Dmax ratio, in conjunction with contemporary treatment strategies and clinical risk models, can further improve prognostic risk stratification.

## 5. Conclusions

MTV and TLG derived from baseline PET/CT appear to be valuable biomarkers for predicting disease burden and treatment response in patients with HL. The observed association between higher metabolic tumor burden and adverse clinical features and lower treatment response rates suggests that these parameters may serve as useful adjuvant biomarkers for baseline risk assessment. However, larger prospective multicenter studies with longer follow-up periods are needed to confirm whether they are prognostic markers for PFS and OS.

## Figures and Tables

**Table 1:** Demographic, Clinical, and Treatment Characteristics of Patients with Hodgkin Lymphoma (*n* = 105).

Age, median (min–max)		33 (18–82)
Gender, *n* (%)	Male	63 (60%)
Histological subtype, *n* (%)	Nodular sclerosis	68 (64.8%)
	Mixed cellularity	29 (27.6%)
	Lymphocyte-rich	5 (4.8%)
	Lymphocyte-depleted	3 (2.8%)
Ann Arbor Stage, *n* (%)	I	6 (5.7%)
	II	50 (47.6%)
	III	17 (16.2%)
	IV	32 (30.5%)
IPS, *n* (%)	Low (0–3)	70 (66.7%)
	High (≥4)	35 (33.3%)
B symptoms, *n* (%)	Absent	56 (53.3%)
	Present	49 (46.7%)
Bulky disease, *n* (%)	Absent	85 (81%)
	Present	20 (19%)
Extranodal involvement, *n* (%)	Absent	73 (69.5%)
	Present	32 (30.5%)
Number of extranodal sites, mean ± SD (*n* = 32)		2 ± 1.7
Interim response, *n* (%) †	CR + PR	93 (93.9%)
	SD + PD	6 (6.1%)
End-of-treatment response, *n* (%) ‡	CR + PR	82 (89.1%)
	SD + PD	10 (10.9%)
Disease progression, *n* (%)	Absent	86 (81.9%)
	Present	19 (18.1%)
Vital status, *n* (%)	Alive	94 (89.5%)
	Death	11 (10.5%)
SUV_mean_, Median (min–max)	4.40 (2–13)	
MTV, Median (min–max)	249 (2–2793)	
TLG, Median (min–max)	1252 (3–11,094)	

CR: Complete response; PR: Partial response; SD: Stable disease; PD: Progressive disease. † ‡ Due to the unavailability of data for certain patients, the evaluation was performed with *n* = 99 patients for interim response and *n* = 92 patients for end-of-treatment response.

**Table 2:** Comparison of Demographic, Clinical, and Treatment Characteristics According to MTV Status (*n* = 105).

		MTV		
		Low (<249) *n* = 52	High (≥249) *n* = 53	*p*-Value
Age, mean ± SD		38.9 ± 15.8	35.6 ± 14.6	0.27 *
Gender, *n* (%)	Female	24 (46.2)	18 (34)	0.20 †
	Male	28 (53.8)	35 (66)	
Ann Arbor Stage, *n* (%)				**<0.001 †**
	I	6 (11.5)	0 (0)	**0.01 §**
	II	32 (61.5)	18 (34)	**0.004 §**
	III	9 (17.3)	8 (15.1)	0.75 §
	IV	5 (9.6)	27 (50.9)	**<0.001 §**
IPS, *n* (%)	Low (0–3)	45 (86.5)	25 (47.2)	**<0.001 †**
	High (≥4)	7 (13.5)	28 (52.8)	
B symptoms, *n* (%)	Absent	37 (71.2)	19 (35.8)	**<0.001 †**
	Present	15 (28.8)	34 (64.2)	
Bulky disease, *n* (%)	Absent	49 (94.2)	36 (67.9)	**0.001 †**
	Present	3 (5.8)	17 (32.1)	
Extranodal involvement, *n* (%)	Absent	47 (90.4)	26 (49.1)	**<0.001** **‡**
	Present	5 (9.6)	27 (50.9)	
İnterim response, *n* (%)	CR + PR	48 (98)	45 (90)	0.20 †
	SD + PD ^a^	1 (2)	5 (10)	
End-of-treatment response, *n* (%)	CR + PR	46 (95.8)	36 (81.8)	**0.04 †**
	SD + PD	2 (4.2)	8 (18.2)	
Disease progression, *n* (%)	Absent	46 (88.5)	40 (75.5)	0.08 †
	Present	6 (11.5)	13 (24.5)	
Vital status, *n* (%)	Alive	47 (90.4)	47 (88.7)	0.78 †
	Death	5 (9.6)	6 (11.3)	

CR: Complete response; PR: Partial response; SD: Stable disease; PD: Progressive disease. * Student’s *t*-test; † Chi-square test; ‡ Mann–Whitney U test; § Z-test for comparison of two proportions. A *p* value < 0.05 was considered statistically significant. ^a^ All six patients with an inadequate interim PET response subsequently underwent treatment escalation from ABVD to escalated BEACOPP.

**Table 3:** Comparison of Demographic, Clinical, and Treatment Characteristics According to TLG Status (*n* = 105).

		TLG		
		Low (<1252) *n* = 52	High (≥1252) *n* = 53	*p*-Value
Age, mean ± SD		39.9 ± 16.4	34.6 ± 13.7	0.08 *
Gender, *n* (%)	Female	24 (46.2)	18 (34)	0.20 †
	Male	28 (53.8)	35 (66)	
Ann Arbor Stage, *n* (%)				**0.004 †**
	I	5 (9.6)	1 (1.9)	0.09 §
	II	30 (57.7)	20 (37.7)	**0.04 §**
	III	9 (17.3)	8 (15.1)	0.75 §
	IV	8 (15.4)	24 (45.3)	**0.001 §**
IPS, *n* (%)	Low (0–3)	45 (86.5)	25 (47.2)	**<0.001 †**
	High (≥4)	7 (13.5)	28 (52.8)	
B symptoms, *n* (%)	Absent	37 (71.2)	19 (35.8)	**<0.001 †**
	Present	15 (28.8)	34 (64.2)	
Bulky disease, *n* (%)	Absent	50 (96.2)	35 (66)	**<0.001 †**
	Present	2 (3.8)	18 (34)	
Extranodal involvement, *n* (%)	Absent	44 (84.6)	29 (54.7)	**0.001** **‡**
	Present	8 (15.4)	24 (45.3)	
Interim response, *n* (%)	CR + PR	48 (100)	45 (88.2)	**0.03 †**
	SD + PD ^a^	0 (0)	6 (11.8)	
End-of-treatment response, *n* (%)	CR + PR	45 (95.7)	37 (82.2)	**0.04 †**
	SD + PD	2 (4.3)	8 (17.8)	
Disease progression, *n* (%)	Absent	45 (86.5)	41 (77.4)	0.22 †
	Present	7 (13.5)	12 (22.6)	
Vital status, *n* (%)	Alive	45 (86.5)	49 (92.5)	0.32 †
	Death	7 (13.5)	4 (7.5)	

CR: Complete response; PR: Partial response; SD: Stable disease; PD: Progressive disease. * Student’s *t*-test; † Chi-square test; ‡ Mann–Whitney U test; § Z-test for comparison of two proportions. A *p* value < 0.05 was considered statistically significant. ^a^ All six patients with an inadequate interim PET response subsequently underwent treatment escalation from ABVD to escalated BEACOPP.

**Table 4:** Comparison of PFS curves (Log-rank test) and hazard ratios with 95% Confidence Interval.

	Disease Progression *n* (%)	Mean PFS (95% CI)	Hazard Ratio (95% CI)	Log- Rank *p* Value
MTV				
≥249	13 (24.53)	41 (35–46)	2.18 (0.88–5.40)	0.09
<249	6 (11.54)	56 (50–62)		
TLG				
≥1252	12 (22.64)	45 (39–51)	1.53 (0.61–3.86)	0.37
<1252	7 (13.46)	54 (48–61)		
Overall	19 (18.10)	49 (43–56)		

CI: Confidence Interval.

**Table 5:** Overall Survival According to MTV and TLG: Kaplan–Meier Analysis and Cox Regression Results.

	Deaths *n* (%)	Mean OS (95% CI)	Hazard Ratio (95% CI)	Log- Rank *p* Value
MTV				
≥249	6 (11.32)	51 (46–55)	1.21 (0.37–3.96)	0.75
<249	5 (9.62)	58 (53–63)		
TLG				
≥1252	4 (7.55)	54 (50–58)	0.56 (0.17–1.85)	0.34
<1252	7 (13.46)	56 (50–61)		
Overall	11 (10.48)	58 (54–61)		

CI: Confidence Interval.

## Data Availability

The data supporting the findings of this study are available from the corresponding author upon reasonable request. The data are not publicly available because they contain information that could compromise patient privacy and are subject to institutional and ethical restrictions.
